# Supplementary material for: Genetic diversity, antifungal evaluation and molecular docking studies of Cu-chitosan nanoparticles as prospective stem rust inhibitor candidates among some Egyptian wheat genotypes
Source: PLoS One. 2021 Nov 12;16(11):e0257959. doi: 10.1371/journal.pone.0257959 (PMC8589204; doi:10.1371/journal.pone.0257959)
Supplement: S1 Table — (DOCX) [file pone.0257959.s001.docx]

| No. | Races | A virulence  (Effective genes) | Virulence  (Ineffective genes) |
| --- | --- | --- | --- |
| 1 | BTSTC | 5, 21, 9e, 7b, 17, 24, 31, 38 | 11, 6, 8a, 9g, 36, 9b, 30, 9a, 9d, 10, Tmp, Mcn |
| 2 | SKTTC | 5, 21, 9e, 11, 36, 9b, 30, 17, 24, 31, 38 | 7b, 6, 8a, 9g, 9a, 9d, 10, Tmp, Mcn |
| 3 | JKGKC | 5, 7b, 11, 36, 30, 17, 9a, 24, 31, 38 | 21,9e, 6, 8a, 9g, 9b, 9d, 10, Tmp, Mcn |
| 4 | SJDCC | 5, 21, 9e, 11, 9g, 36, 9b, 17, 9a, 9d, 10, 24, 31, 38 | 7b, 6, 8a, 30, Tmp, Mcn |
| 5 | DKTTC | 5, 21, 7b, 11, 36, 9b, 30, 17, 24, 31, 38 | 9e, 6, 8a, 9g, 9a, 9d, 10, Tmp, Mcn |
| 6 | PKTTC | 21, 11, 36, 9b, 30, 17, 24, 31, 38 | 5, 9e, 7b, 6, 8a, 9g, 9a, 9d, 10, Tmp, Mcn |
| 7 | PKTTH | 21, 11, 36, 9b, 30, 17, 24, 31, 38 | 5, 9e, 7b, 6, 8a, 9g, 9a, 9d, 10, Tmp, Mcn |
| 8 | PCTKC | 21, 11, 6, 8a, 36, 9b, 30, 17, 9a, 24, 31, 38 | 5, 9e, 7b, 9g, 9d, 10, Tmp, Mcn |
| 9 | STKTC | 5, 21, 9e, 36, 24, 31, 38 | 7b, 11, 6, 8a, 9g, 9b, 30, 17, 9a, 9d, 10, Tmp, Mcn |
| 10 | TKTPC | 11, 36, 9b, 30, 17, 9d, 24, 31, 38 | 5, 21, 9e, 7b, 6, 8a, 9g, 9a, 10, Tmp, Mcn |
| 11 | TKPTC | 11, 9b, 24, 31, 38 | 5, 21, 9e, 7b, 6, 8a, 9g, 36, 30, 17, 9a, 9d, 10, Tmp, Mcn |
| 12 | TKSTC | 11, 17, 24, 31, 38 | 5, 21, 9e, 7b, 6, 8a, 9g, 36, 9b, 30, 9a, 9d, 10, Tmp, Mcn |
| 13 | TKTTC | 11, 36, 9b, 30, 17, 24, 31, 38 | 5, 21, 9e, 7b, 6, 8a, 9g, 9a, 9d, 10, Tmp, Mcn |
| 14 | TTKSC | 36, Tmp, 24, 31, 38 | 5, 21, 9e, 7b, 11, 6, 8a, 9g, 9b, 30, 17, 9a, 9d, 10, Mcn |
| 15 | TTTSK | 36, 9b, 30, 17, Tmp, 24, | 5, 21, 9e, 7b, 11, 6, 8a, 9g, 9a, 9d, 10, Mcn |
| 16 | PKTTH | 21, 11, 36, 9b, 30, 17, 24, 38 | 5, 9e, 7b, 6, 8a, 9g, 9a, 9d, 10, Tmp, Mcn |
| 17 | PCTKC | 21, 11, 6, 8a, 36, 9b, 30, 17, 9a, 24, 31, 38 | 5, 9e, 7b, 9g, 9d, 10, Tmp, Mcn |
| 18 | STKTC | 5, 21, 9e, 36, 24, 31, 38 | 7b, 11, 6, 8a, 9g, 9b, 30, 17, 9a, 9d, 10, Tmp, Mcn |
| 19 | TKTPC | 11, 36, 9b, 30, 17, 9d, 24, 31, 38 | 5, 21, 9e, 7b, 6, 8a, 9g, 9a, 10, Tmp, Mcn |
| 20 | TKPTC | 11, 9b, 24, 31, 38 | 5, 21, 9e, 7b, 6, 8a, 9g, 36, 30, 17, 9a, 9d, 10, Tmp Mcn |

Table (S1). The a virulence/virulence pattern of 20 *Puccinia graminis f.sp. tritici* pathotypes used to study stem rust resistance of eighteen Egyptian wheat genotypes
