# Supplementary material for: Genetic diversity, antifungal evaluation and molecular docking studies of Cu-chitosan nanoparticles as prospective stem rust inhibitor candidates among some Egyptian wheat genotypes
Source: PLoS One. 2021 Nov 12;16(11):e0257959. doi: 10.1371/journal.pone.0257959 (PMC8589204; doi:10.1371/journal.pone.0257959)
Supplement: S2 Table — (DOCX) [file pone.0257959.s002.docx]

Table (S2). A virulence/virulence, based on seedling reaction for 5 isolates leaf rust in 2019/2020 season.

| No. | Races | A virulence  (Effective genes) | Virulence  (Ineffective genes) |
| --- | --- | --- | --- |
| 1 | DTJHC | 1, 2a, 3ka, 30, 10, 21, 14b, 15, 36, | 2c, 3, 9, 16, 24, 26,11, 17, 18, 2b, 2b, 42 |
| 2 | JKGTC | 1, 3, 9, 3ka, 17, 30, 14b, 15, 36 | 2a, 2c, 16, 24, 26, 11, 10, 18, 21, 2b, 42 |
| 3 | TJTPC | 9, 26, 18, 14b, 15, 36 | 1, 2a, 2c, 3, 16, 24, 3ka, 11, 17, 30, 10, 21, 2b, 42 |
| 4 | TKKTC | 9, 3ka, 14b, 15, 36 | 1, 2a, 2c, 3, 16. 24, 26, 11, 17, 30, 10, 18, 21, 2b, 42 |
| 5 | PKPTC | 2a, 9, 11, 14b, 15, 36 | 1, 2c, 3, 16, 24, 26, 3ka, 17, 30, 10, 18, 21, 2b, 42 |
