# Supplementary material for: Genetic diversity, antifungal evaluation and molecular docking studies of Cu-chitosan nanoparticles as prospective stem rust inhibitor candidates among some Egyptian wheat genotypes
Source: PLoS One. 2021 Nov 12;16(11):e0257959. doi: 10.1371/journal.pone.0257959 (PMC8589204; doi:10.1371/journal.pone.0257959)
Supplement: S3 Table — (DOCX) [file pone.0257959.s003.docx]

**Table S3. Genetic similarity matrix among the 18 wheat genotypes based on the Dice coefficient generated from SCOT marker.**

| Genotypes | 1 | 2 | 3 | 4 | 5 | 6 | 7 | 8 | 9 | 10 | 11 | 12 | 13 | 14 | 15 | 16 | 17 | 18 |
| --- | --- | --- | --- | --- | --- | --- | --- | --- | --- | --- | --- | --- | --- | --- | --- | --- | --- | --- |
| Gemmeiza 11 | 1.00 |  |  |  |  |  |  |  |  |  |  |  |  |  |  |  |  |  |
| Gemmeiza 12 | 0.69 | 1.00 |  |  |  |  |  |  |  |  |  |  |  |  |  |  |  |  |
| Sids 12 | 0.67 | 0.80 | 1.00 |  |  |  |  |  |  |  |  |  |  |  |  |  |  |  |
| Misr 1 | 0.77 | 0.69 | 0.69 | 1.00 |  |  |  |  |  |  |  |  |  |  |  |  |  |  |
| Misr 2 | 0.65 | 0.73 | 0.67 | 0.77 | 1.00 |  |  |  |  |  |  |  |  |  |  |  |  |  |
| Misr 3 | 0.61 | 0.69 | 0.65 | 0.69 | 0.71 | 1.00 |  |  |  |  |  |  |  |  |  |  |  |  |
| Giza 168 | 0.68 | 0.56 | 0.53 | 0.70 | 0.62 | 0.64 | 1.00 |  |  |  |  |  |  |  |  |  |  |  |
| Giza 171 | 0.53 | 0.58 | 0.48 | 0.66 | 0.62 | 0.68 | 0.60 | 1.00 |  |  |  |  |  |  |  |  |  |  |
| Sakha 94 | 0.54 | 0.59 | 0.55 | 0.66 | 0.64 | 0.68 | 0.58 | 0.71 | 1.00 |  |  |  |  |  |  |  |  |  |
| Sakha 95 | 0.44 | 0.54 | 0.55 | 0.52 | 0.65 | 0.49 | 0.47 | 0.51 | 0.63 | 1.00 |  |  |  |  |  |  |  |  |
| Beni Sweif 7 | 0.53 | 0.49 | 0.55 | 0.57 | 0.55 | 0.50 | 0.50 | 0.50 | 0.57 | 0.53 | 1.00 |  |  |  |  |  |  |  |
| Shandaweel 1 | 0.38 | 0.50 | 0.48 | 0.50 | 0.54 | 0.51 | 0.47 | 0.51 | 0.63 | 0.74 | 0.51 | 1.00 |  |  |  |  |  |  |
| Giza 164 | 0.58 | 0.55 | 0.53 | 0.62 | 0.58 | 0.56 | 0.54 | 0.64 | 0.66 | 0.63 | 0.54 | 0.61 | 1.00 |  |  |  |  |  |
| Sakha 69 | 0.54 | 0.59 | 0.57 | 0.58 | 0.58 | 0.61 | 0.47 | 0.62 | 0.62 | 0.61 | 0.51 | 0.57 | 0.84 | 1.00 |  |  |  |  |
| Giza 160 | 0.50 | 0.52 | 0.51 | 0.63 | 0.57 | 0.59 | 0.53 | 0.65 | 0.71 | 0.57 | 0.53 | 0.62 | 0.74 | 0.74 | 1.00 |  |  |  |
| Beni Sweif 4 | 0.43 | 0.46 | 0.46 | 0.49 | 0.49 | 0.50 | 0.47 | 0.56 | 0.58 | 0.59 | 0.48 | 0.63 | 0.66 | 0.67 | 0.74 | 1.00 |  |  |
| Sohag 4 | 0.48 | 0.39 | 0.46 | 0.51 | 0.42 | 0.45 | 0.52 | 0.45 | 0.44 | 0.48 | 0.58 | 0.47 | 0.60 | 0.55 | 0.57 | 0.59 | 1.00 |  |
| Sohag 5 | 0.45 | 0.54 | 0.55 | 0.58 | 0.54 | 0.61 | 0.51 | 0.59 | 0.62 | 0.66 | 0.50 | 0.64 | 0.75 | 0.74 | 0.73 | 0.74 | 0.55 | 1.00 |
