# Supplementary material for: Genetic diversity, antifungal evaluation and molecular docking studies of Cu-chitosan nanoparticles as prospective stem rust inhibitor candidates among some Egyptian wheat genotypes
Source: PLoS One. 2021 Nov 12;16(11):e0257959. doi: 10.1371/journal.pone.0257959 (PMC8589204; doi:10.1371/journal.pone.0257959)
Supplement: S5 Table — (DOCX) [file pone.0257959.s005.docx]

**Table S5. Genetic similarity matrix among the 18 wheat genotypes based on the Dice coefficient generated from combined data.**

| **Genotypes** | **1** | **2** | **3** | **4** | **5** | **6** | | | **7** | **8** | **9** | **10** | **11** | **12** | **13** | **14** | **15** | **16** | **17** | **18** |
| --- | --- | --- | --- | --- | --- | --- | --- | --- | --- | --- | --- | --- | --- | --- | --- | --- | --- | --- | --- | --- |
| **Gemmeiza 11** | 1.00 |  |  |  |  |  | | |  |  |  |  |  |  |  |  |  |  |  |  |
| **Gemmeiza 12** | 0.78 | 1.00 |  |  |  |  | | |  |  |  |  |  |  |  |  |  |  |  |  |
| **Sids 12** | 0.74 | 0.87 | 1.00 |  |  |  | | |  |  |  |  |  |  |  |  |  |  |  |  |
| **Misr 1** | 0.80 | 0.74 | 0.75 | 1.00 |  |  | | |  |  |  |  |  |  |  |  |  |  |  |  |
| **Misr 2** | 0.76 | 0.74 | 0.72 | 0.82 | 1.00 | | |  |  |  |  |  |  |  |  |  |  |  |  |  |
| **Misr 3** | 0.75 | 0.78 | 0.77 | 0.79 | 0.81 | | 1.00 | |  |  |  |  |  |  |  |  |  |  |  |  |
| **Giza 168** | 0.78 | 0.71 | 0.69 | 0.80 | 0.79 | | 0.81 | | 1.00 |  |  |  |  |  |  |  |  |  |  |  |
| **Giza 171** | 0.68 | 0.74 | 0.72 | 0.74 | 0.72 | | 0.80 | | 0.75 | 1.00 |  |  |  |  |  |  |  |  |  |  |
| **Sakha 94** | 0.68 | 0.70 | 0.70 | 0.77 | 0.74 | | 0.78 | | 0.74 | 0.81 | 1.00 |  |  |  |  |  |  |  |  |  |
| **Sakha 95** | 0.61 | 0.68 | 0.70 | 0.68 | 0.71 | | 0.67 | | 0.66 | 0.72 | 0.77 | 1.00 |  |  |  |  |  |  |  |  |
| **Beni Sweif 7** | 0.63 | 0.61 | 0.66 | 0.67 | 0.65 | | 0.66 | | 0.66 | 0.67 | 0.71 | 0.71 | 1.00 |  |  |  |  |  |  |  |
| **Shandaweel 1** | 0.53 | 0.61 | 0.63 | 0.61 | 0.63 | | 0.65 | | 0.61 | 0.65 | 0.71 | 0.77 | 0.66 | 1.00 |  |  |  |  |  |  |
| **Giza 164** | 0.63 | 0.63 | 0.62 | 0.65 | 0.64 | | 0.64 | | 0.63 | 0.69 | 0.71 | 0.70 | 0.65 | 0.71 | 1.00 |  |  |  |  |  |
| **Sakha 69** | 0.60 | 0.63 | 0.64 | 0.65 | 0.65 | | 0.67 | | 0.60 | 0.66 | 0.68 | 0.69 | 0.66 | 0.72 | 0.85 | 1.00 |  |  |  |  |
| **Giza 160** | 0.60 | 0.60 | 0.61 | 0.66 | 0.64 | | 0.65 | | 0.63 | 0.67 | 0.73 | 0.66 | 0.65 | 0.74 | 0.80 | 0.85 | 1.00 |  |  |  |
| **Beni Sweif 4** | 0.54 | 0.56 | 0.57 | 0.58 | 0.57 | | 0.59 | | 0.58 | 0.62 | 0.65 | 0.66 | 0.61 | 0.70 | 0.76 | 0.76 | 0.81 | 1.00 |  |  |
| **Sohag 4** | 0.55 | 0.53 | 0.58 | 0.59 | 0.56 | | 0.57 | | 0.60 | 0.60 | 0.57 | 0.60 | 0.64 | 0.63 | 0.71 | 0.68 | 0.69 | 0.72 | 1.00 |  |
| **Sohag 5** | 0.53 | 0.58 | 0.62 | 0.60 | 0.58 | | 0.62 | | 0.57 | 0.64 | 0.67 | 0.70 | 0.62 | 0.74 | 0.76 | 0.78 | 0.78 | 0.78 | 0.66 | 1.00 |
