# Supplementary material for: Genetic diversity, antifungal evaluation and molecular docking studies of Cu-chitosan nanoparticles as prospective stem rust inhibitor candidates among some Egyptian wheat genotypes
Source: PLoS One. 2021 Nov 12;16(11):e0257959. doi: 10.1371/journal.pone.0257959 (PMC8589204; doi:10.1371/journal.pone.0257959)
Supplement: S6 Table — (DOCX) [file pone.0257959.s006.docx]

**Table S6. Sequence identity, Query coverage and QMEAN scores of templates structure for homology model building of MAPK1 and PgMAPK proteins of *P. triticina* and *P. graminis tritici* through BLAST against RCSB PDB**

| Fungi | Protein / accession number | Templates (PDB id) | Chain | Query coverage (%) | QMEAN | % of identity | Resolution (Å) |
| --- | --- | --- | --- | --- | --- | --- | --- |
| *P. graminis tritici* | PgMAPK 10532062 | [2VKN](file:///G:\Dr%20hanaa%20nano\protien\P\PGTG_01_2021-01-06\PGTG_01_2021-01-06\model\01\templates\2vkn.1.A.pdb.gz) | A | 0.12 | -0.69 | 56.14 | 2.05 |
| *P. triticina* | MAPK1 AAY89655.1 | [2FA2](file:///G:\Dr%20hanaa%20nano\protien\mpk1%20leaf\MAPK1_05_2021-01-%20leaf\MAPK1_05_2021-01-04\model\05\templates\2fa2.1.A.pdb.gz) | A | 0.84 | -1.53 | 56.14 | 2.85 |
